# Coloration of Flowers by Flavonoids and Consequences of pH Dependent Absorption

**DOI:** 10.3389/fpls.2020.600124

**Published:** 2021-01-08

**Authors:** Doekele G. Stavenga, Hein L. Leertouwer, Bettina Dudek, Casper J. van der Kooi

**Affiliations:** ^1^Zernike Institute for Advanced Materials, University of Groningen, Groningen, Netherlands; ^2^Research Group Biosynthesis/NMR, Max Planck Institute for Chemical Ecology, Jena, Germany; ^3^Groningen Institute for Evolutionary Life Sciences, University of Groningen, Groningen, Netherlands

**Keywords:** flavonol, anthocyanin, nudicaulin, carotenoid, pollination, *Papaver*, *Mandevilla*

## Abstract

Flavonoid pigments are key determinants of flower colors. As absorption spectra of flavonoids are known to be severely pH-dependent, cellular pH will play a crucial role in flower coloration. The flavonoids are concentrated in the vacuoles of the flowers’ epidermal cells, and thus the pigments’ absorption spectra are modulated by the vacuolar pH. Here we study the pH dependence of flavonoid absorption spectra in extracts from flowers of two poppy species *Papaver dubium* (red) and *Meconopsis cambrica* (orange), and a white and red *Mandevilla sanderi* variety. In the red poppy and *Mandevilla* flowers, absorption spectra of the cyanidin- and pelargonidin-based anthocyanins peak in the blue-green-wavelength range at low pH, but exhibit a distinct bathochromic shift at higher pH. This shift to longer wavelengths is not found for the blue-absorbing nudicaulin derivatives of *M. cambrica*, which have a similar absorption spectrum at low and high pH. The pH-dependent absorption changes of the white *M. sanderi*’s flavonoid remained restricted to the UV. An analysis of the spectra with logistic functions suggests that the pH-dependent characteristics of the basic states of flavonols and anthocyanins are related. The implications of tuning of pH and pigment absorption spectra for studies on flower color evolution are discussed.

## Introduction

The plant kingdom harbors a remarkable diversity in flower colors. This colorful richness arose mostly because their coloration aid flowers in attracting pollinators, thereby enhancing the plants’ reproductive success (van der Kooi and Ollerton, 2020). The visibility of a flower, and thus its attractiveness, is principally determined by the wavelength dependence of the fraction of incident light that is back-scattered. Whereas the backscattering is determined by the inhomogeneous structuring of the petals, the color of the backscattered light mainly depends on the flower’s pigmentation (van der Kooi et al., 2016). Presumably, the pigment type is broadly correlated with pollination ecology, with flower colors being tuned to the visual system of the pollinators (Harborne and Smith, 1978; Chittka and Menzel, 1992; Brouillard and Dangles, 1994; de Camargo et al., 2019; Shrestha et al., 2019; van der Kooi et al., 2019).

The most common flower pigments are the carotenoids and flavonoids (Grotewold, 2006). The blue-absorbing carotenoids, e.g. zeaxanthin, carotene, and lutein, create yellow tissues, and the green-absorbing astaxanthin causes a red color (Yuan et al., 2002; Grotewold, 2006; Shafaa et al., 2007; Rasmussen et al., 2012). Of the flavonoids, the flavonols absorb virtually exclusively in the UV and thus cause white to pale-yellow colors. Widespread flower pigments are the spectrally variably absorbing anthocyanins, which can confer red, blue, or purple colors to plant tissues (e.g., Markham, 1982; Brouillard and Dangles, 1994). The anthocyanin pigments, glycosides of anthocyanidin aglycons, are secondary metabolites of land plants that can be biochemically detected in species as ancient as mosses (Bendz et al., 1962; Koes et al., 1994; Rausher, 2006; Campanella et al., 2014). The three major anthocyanin pigment types that are found in terrestrial plants are cyanidin-3-glycoside (brick red), pelargonidin-3-glycoside (orange/red), and delphinidin-3-glycoside (blue/purple), of which cyanidin is more present in primitive families, while delphinidin is restricted to the more highly evolved angiosperm plant families (Harborne and Williams, 2000).

Anthocyanin pigments are water soluble and concentrated in the epidermal cells, where the pigments are most effective in creating an intense coloration (van der Kooi et al., 2016). Importantly, the anthocyanins occur in the vacuoles, which often have a quite acidic pH (Pourcel et al., 2010; Passeri et al., 2016). The acidic vacuolar pH is probably intimately related to the severe pH dependence of the flavonoid absorption spectra (e.g., Jurasekova et al., 2014; Tang et al., 2019). The factors and mechanisms determining the vacuolar pH thus play a central role in flower coloration and are important for pollination.

We previously investigated how pigment absorption and scattering together determine the colors of the flowers of a few poppy species (van der Kooi and Stavenga, 2019; Dudek et al., 2020; Martínez-Harms et al., 2020). We thus found that the flowers of the common poppy (*Papaver rhoeas*) in the Middle East exhibit a low UV-reflectance due to a considerable amount of the flavonols kaempferol and quercetin, but those in Germany have much less of the flavonols and hence have a distinct UV reflectance (Dudek et al., 2020). The anthocyanins responsible for the bright red colors of *P. rhoeas* and the related long-headed poppy (*Papaver dubium*) are cyanidin and pelargonidin glycosides (Scott-Moncrieff, 1936; Acheson et al., 1956, 1962; Harborne, 1958; Dudek et al., 2020). The *in situ* absorbance spectra of the red flowers of the European *P. rhoeas* and *P. dubium* are very similar, but *P. dubium* appears to have a lower concentration of pelargonidin (Figure 6A in van der Kooi and Stavenga, 2019).

Previous investigations on the pH dependence of pigments mostly concentrated on single anthocyanins *in vitro*, or studied the influence of pH on the complex mechanisms of blue flower coloration (Mazza and Brouillard, 1987; Yoshida et al., 2009). Here we investigate how the absorbance spectra of the flavonoid pigments depend on pH. We chose two sets of species/varieties that are roughly similar in anatomy and backscattering, but differ in coloration due to different pigmentation. We thus compare flowers of *P. dubium* (red), the related Welsh poppy *Meconopsis cambrica* (orange), and two different-colored varieties of *Mandevilla sanderi* (also known as *Dipladenia*). We conclude that the vacuolar pH plays a crucial role for realizing strongly colored flowers with anthocyanins that are attractive for pollinators and discuss how pH is important for studies on the evolution of flower coloration.

## Materials and Methods

### Plant Material and Photography

Long-headed poppies, *P. dubium*, were collected at road sides in Groningen, Netherlands. The *M. sanderi* plants, “Sundaville White” and “Sundaville Red,” hereafter abbreviated as White and Red, were obtained from local suppliers. Welsh poppies, *M. cambrica*, were taken from a local garden. Macro-photographs of the flowers were made with a Canon EOS 7D.

### Spectrophotometry of Flower Lobes and Extracts

Reflectance spectra of flower lobes were performed with a bifurcated reflection probe. The light source was a deuterium-halogen lamp [AvaLight-D(H)-S] and the spectrometer an AvaSpec-2048 (Avantes, Apeldoorn, Netherlands). Pigments were extracted from 1 to 5 cm^2^-sized petal or lobe pieces in a ∼40 ml solution of 50:1 methanol: 1 M hydrochloric acid (MeOH and HCL purchased from Sigma Aldrich, Steinheim, Germany). Absorbance spectra of the extracted pigments were measured immediately after the extraction in 10 mm light path quartz cuvettes at room temperature. The pH of the extract, measured with a calibrated pH meter, was modified by adding adequate amounts of KOH solution. The pH-dependence of the absorbance (or optical density *D*), was evaluated at a few different wavelengths.

When a medium contains more than one pigment, and the concentration of one pigment changes due to the action of some agent (e.g., a change in pH), the different components of the process of the pigment changes can be separated by subtracting the constant background absorbance caused by the other pigments. When the absorbance increased with increasing pH the values were approximated by

(1a) $$D=\sum_{i}^{n}A_{i}\,F_{i}+B$$

and when the absorbance decreased with increasing pH by

(1b) $$D=\sum_{i}^{n}A_{i}\,\left(1-F_{i}\right)+B$$

Here *F*_*i*_ is a logistic function, with parameter pK*_*i*_*

(1c) $$F_{i}=1/\left[1+10^{-\left(\text{pH}-{\text{pK}}_{i}\right)}\right]$$

which accounts for *n* = 1 or 2 pH-dependent components with amplitude *A*_*i*_ and background *B*. We evaluated the absorbance changes at those critical wavelengths where the changes of pigment states were large. The pK-values of the components resulted from fits of Eq. 1 to the measurements.

## Results

### Papaver dubium

**FIGURE 1:**
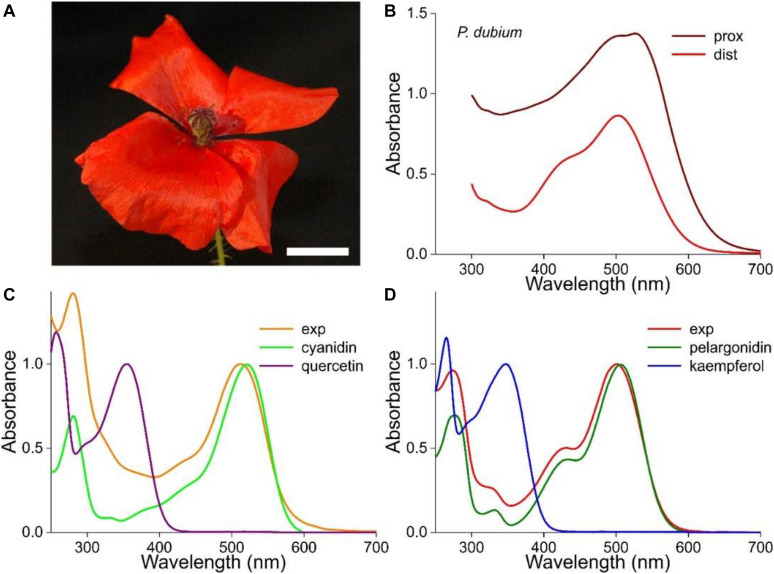
Absorbance spectra of the long-headed poppy *Papaver dubium*. **(A)** A *P. dubium* flower; scale bar 1 cm. **(B)** Absorbance spectra of a proximal and distal petal area (from van der Kooi and Stavenga, 2019). **(C)** Absorbance spectrum of a flower extract (exp; normalized spectrum for pH = 2.5 of Figure 2A) compared with the spectrum of isolated cyanidin; the spectrum of quercetin is added. **(D)** Absorbance spectrum measured of another flower extract (exp; normalized spectrum for pH = 2.4 of Figure 3A) compared with the spectrum of isolated pelargonidin; the spectrum of kaempferol is added (flavonoid spectra from Dudek et al., 2020).

The flowers of the longheaded poppy, *P. dubium*, display an almost homogeneous, bright-red color (Figure 1A). However, absorbance spectra measured at the distal and proximal flower areas differ distinctly in amplitude as well as shape, which shows that the pigmentation causing the red color is not due to one pigment that is distributed inhomogeneously (Figure 1B).

We investigated the pigments in methanol extracts from different flowers of *P. dubium*, which yielded bright red solutions. Figures 1C,D show two exemplary cases where the measured absorbance spectra (exp) have rather different shapes, with peak wavelengths at 512 and 501 nm, respectively. These experimental spectra closely resemble the absorption spectra of cyanidin and pelargonidin glycosides, the two fundamental anthocyanidins that were isolated from flowers of the common poppy *P. rhoeas*, which have peak wavelengths 521 and 507 nm, respectively (Figures 1C,D; from Dudek et al., 2020). We hence conclude that *P. dubium* flowers contain variable amounts of cyanidin and pelargonidin derivatives, in agreement with previous studies (Scott-Moncrieff, 1936; Acheson et al., 1956, 1962).

We have added to Figures 1C,D the absorption spectra of quercetin and kaempferol glycosides, because they were also identified in *P. rhoeas* flowers (Dudek et al., 2020). These flavonols have the same hydroxylation pattern and are derived from the same precursor as the two anthocyanins. The contribution of the two flavonols to the measured absorbance spectra is clearly minor, in agreement with reflectance and transmittance spectra measured on intact *P. dubium* flowers, which also indicated that absorption in the UV is very moderate (see Figures 1E,F of van der Kooi and Stavenga, 2019).

**FIGURE 2:**
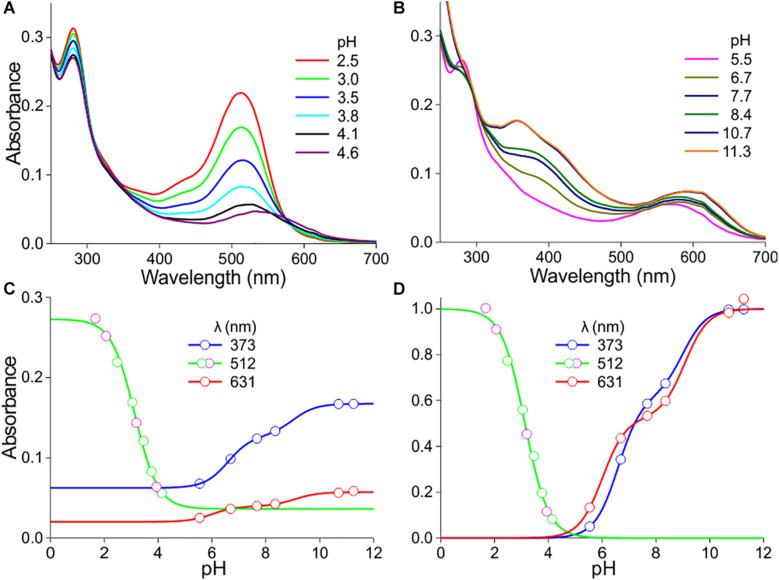
pH dependence of *P. dubium* pigments. **(A)** Absorbance spectra of extract for pH < 5. **(B)** Absorbance spectra for pH > 5. **(C)** Absorbance values derived from **(A,B)** at a few wavelengths, fitted with logistic functions. **(D)** The data of **(C)** with background subtracted and normalized [green symbols in **(C,D)** derived from **(A)**, magenta symbols from another measurement series].

**FIGURE 3:**
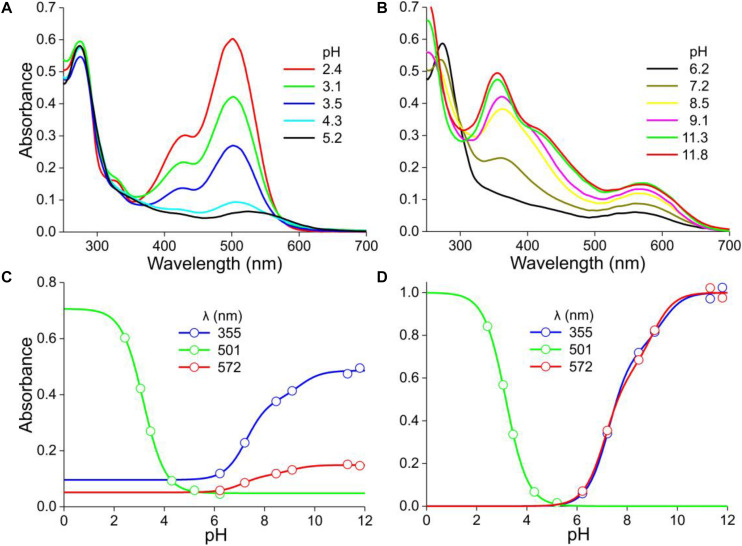
Absorbance spectra of another *P. dubium* flower extract at various pH. **(A)** Absorbance spectra for pH < 6. **(B)** Absorbance spectra for pH > 6. **(C)** Absorbance values derived from **(A,B)** at a few wavelengths, fitted with logistic functions. **(D)** The data of **(C)** with background subtracted and normalized.

The anthocyanin spectra severely depend on pH (Figures 2, 3). Figures 2A,B show how the absorbance spectra of a flower extract (the case of the experimental spectrum of Figure 1C) change when the pH gradually increases. For pH < 5, the absorbance in the blue-green wavelength range decreases about proportionally (Figure 2A). The absorbance values assessed at the peak wavelength, 512 nm, are well approximated with a one-component logistic function (using Eqs 1b,c), yielding pK = 3.1 (Figure 2C), which suggests the pH-dependent transition of two pigment states.

For pH > 5, the absorbance in the ultraviolet (UV-A; peak wavelength ∼373 nm) as well as orange/red wavelength range (peak wavelength ∼631 nm) steadily increases. The absorbance values of the pH-dependent spectra at 373 nm approximated with a two-component logistic function (using Eqs 1a,c) yielded pK-values of 6.6 and 9.0. The absorbance values at 631 nm could also be well described by a two-component logistic function, yielding similar pK values: pK = 6.0 and 9.1 (Figure 2C). Subtraction of the background and subsequently normalizing the data of Figure 2C yielded Figure 2D.

Figures 3A,B show how the absorbance spectra of another flower extract (that of the experimental spectrum of Figure 1D) change when the pH gradually increases. For pH < 5, the absorbance values at the peak wavelength, 501 nm, fitted with a one-component logistic function, yielded pK = 3.2 (Figure 3C), very similar to the pK = 3.1 of Figure 2C. For pH > 5, fitting a two-component logistic function to the absorbance values at 355 nm yielded pK = 7.3 and 9.4, whilst those at 572 nm produced pK = 7.1 and 9.0 (Figure 3C). Subtraction of the background and subsequently normalizing the data of Figure 3C yielded Figure 3D.

**FIGURE 4:**
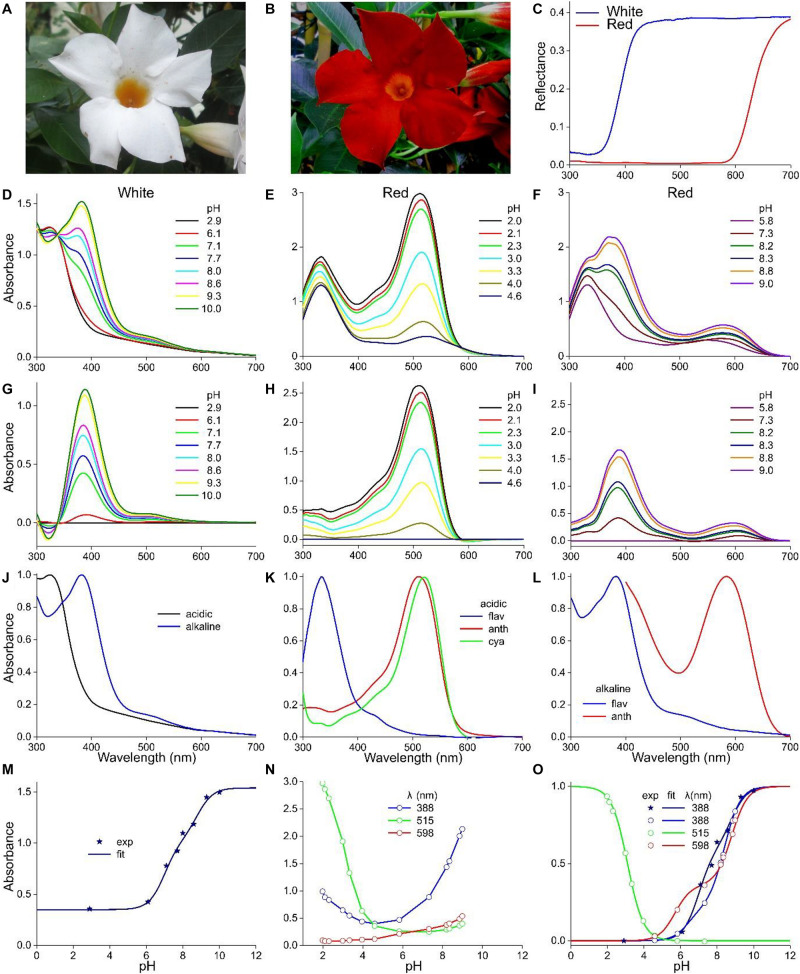
*Mandevilla sanderi* flowers and absorbance spectra of extracted pigment at various pH values. **(A)** Flower of a White Sundaville morph with white lobe and yellow tube. **(B)** Flower of a Red Sundaville morph with red lobe and orange tube. **(C)** Reflectance spectra of the lobes of the flowers of panels **(A,B)**. **(D)** Absorbance spectra of pigment extracted from a White lobe. **(E)** Absorbance spectra of pigment extracted from a Red lobe, measured at low pH values. **(F)** as **(E)**, but spectra measured at higher pH-values. **(G)** Absorbance difference spectra obtained by subtracting the spectrum for pH = 2.9 from the spectra of panel **(D)**. **(H)** Absorbance difference spectra obtained by subtracting the spectrum for pH = 4.6 from the spectra of panel **(E)**. **(I)** Absorbance difference spectra obtained by subtracting the spectrum for pH = 5.8 from the spectra of panel **(F)**. **(J)** Normalized absorbance spectra of the White lobe in the acid and alkaline state. **(K)** Normalized absorbance spectra of flavonols and anthocyanins of the Red lobe in the acid state, together with the cyanidin spectrum of Figure 1C. **(L)** Normalized absorbance spectra of the flavonols and anthocyanins of the Red lobe in the alkaline state. **(M)** Absorbance values at 388 nm of the spectra of panel **(D)** as a function of pH (symbols), fitted with Eq. 1 (solid line). **(N)** Absorbance values at 388, 515, and 598 nm of the spectra of panels **(E,F)** as a function of pH. **(O)** Background subtracted and normalized absorbance values of panels **(M,N)** together with fits.

The pK-values of the logistic functions in Figures 2C,D, 3C,D appear to be very similar, which suggests that the same pH-dependent structural changes govern the absorbance changes of cyanidin and pelargonidin upon decreasing acidity or increasing alkalinity (Pina et al., 2012). To further investigate this, we performed the same experimental approach on extracts obtained from flowers of the White and Red Sundaville morphs of *M. sanderi* plants (Figures 4A,B).

### Mandevilla sanderi

The flower lobes of *M. sanderi* “White Sundaville” are brightly white colored (Figure 4A), which might suggest the absence of pigments, but reflectance spectra show that this is certainly not the case (Figure 4C). The lobe’s reflectance was indeed high throughout the visible wavelength range, but the low reflectance in the UV revealed the presence of UV-absorbing pigments; a common feature in (to humans) white flowers (Chittka et al., 1994; Kevan et al., 1996). Methanol extracts of the White flower’s lobes had for pH < 5 virtually identical absorbance spectra, with peak wavelength ∼320 nm. At pH > 5, with increasing pH the absorbance increased and shifted bathochromically, peaking at 385 nm (Figure 4D). An isosbestic point at 337 nm suggested the existence of two pigment states with different absorption spectra. Absorbance difference spectra calculated by subtracting the absorbance spectrum of pH = 2.9 from the measured spectra indeed had a very similar shape (Figure 4G). Figure 4J shows normalized spectra of the low-pH (acid) and high-pH (alkaline) states. To further characterize this, we evaluated the absorbance spectra of Figure 4D at λ = 388 nm, the peak wavelength of the difference spectra (asterisks in Figure 4M). The data could only be well-fitted with a two-component logistic function (Eqs 1a,c), which yielded pK-values 6.9 and 8.8, thus indicating that more than two pigment states are involved.

The pigments extracted from the flower lobes of *M. sanderi* “Red Sundaville” (Figure 4B) behaved rather differently. At low pH-values, the absorbance spectrum featured two bands, peaking at 330 nm and 515 nm. Increasing the pH-value from 2.0 to 4.6 caused a severe drop of the main band in the green wavelength range (Figure 4E). To analyze this pH-dependent change, we calculated again absorbance difference spectra, by subtracting the absorbance spectrum measured at pH = 4.6 from the absorbance spectra measured at pH < 5. The resulting difference spectra were about proportional to each other, suggesting again a proportional change in a pigment state depending on the pH (Figure 4H). The average of the spectra of Figure 4H, which is shown normalized in Figure 4K (anth), closely resembles the cyanidin spectrum of Figure 1C (Figure 4K, cya).

We analyzed the pH-dependence of this pigment state by assessing the absorbance at its peak wavelength (515 nm) as a function of pH (Figure 4N). The pH-dependence could be well fitted with a single-component logistic function (Eqs 1b,c), yielding pK = 3.1, identical to the *P. dubium* case of Figure 2. This confirms that cyanidin is the prominent anthocyanin that determines the coloration of *M. sanderi* “Red Sundaville.” Yet, Figures 4E,H clearly show that the flowers contain, in addition to the blue-green absorbing cyanidin, a UV-absorbing pigment. We, therefore, normalized the spectra of Figure 4E, subtracted the average anthocyanin spectrum (Figure 4K, anth) and then normalized the results. This yielded an absorbance spectrum peaking at 325 nm, which we tentatively assume to be due to flavonols (Figure 4K, flav). However, quite possibly the pigment may actually be another flavonoid, e.g., a flavone (Markham, 1982).

When the pH increased to above five, an absorbance band peaking around 600 nm gradually emerged. In addition, the UV-band shifted bathochromically, resulting in a prominent band peaking at 380 nm (Figure 4F), which resembles the absorbance band peaking at 380 nm that emerged with pH > 5 in the case of the White lobe’s extract (Figure 4D). We furthermore analyzed the set of spectra again by subtracting the absorbance spectrum measured at pH = 5.8 from the other spectra measured at pH > 5, which yielded absorbance difference spectra with two absorbance bands with peak wavelengths 388 and 598 nm (Figure 4I).

We subsequently evaluated the absorbance spectra of Figures 4E,F at these peak wavelengths (Figure 4N). The absorbance values at 388 nm as a function of pH showed a biphasic behavior, with first a decline between pH = 2 and 5 (Figure 4N). This absorbance decline is apparently due to the fall in the cyanidin concentration (Figure 4H), as the data for pH < 5 were well approximated by Eqs 1b,c, using pK = 3.1. For pH > 5, the 388 nm data could be well fitted with Eqs 1a,c (*n* = 2), yielding pK = 6.4 and 8.4.

We finally analyzed the pH dependence of the long-wavelength absorbance band by estimating the absorbance at 598 nm as a function of pH and fitting Eqs 1a,c to the data, which yielded pK = 5.7 and 8.8 (Figure 4N). Subtracting the background and normalizing the data as before yielded Figure 4O. Taking the calculated pH-dependencies into account, we finally analyzed the difference spectra of Figure 4I, which produced the normalized absorbance spectrum of a UV- and a red-absorbing pigment (Figure 4L).

### Meconopsis cambrica

**FIGURE 5:**
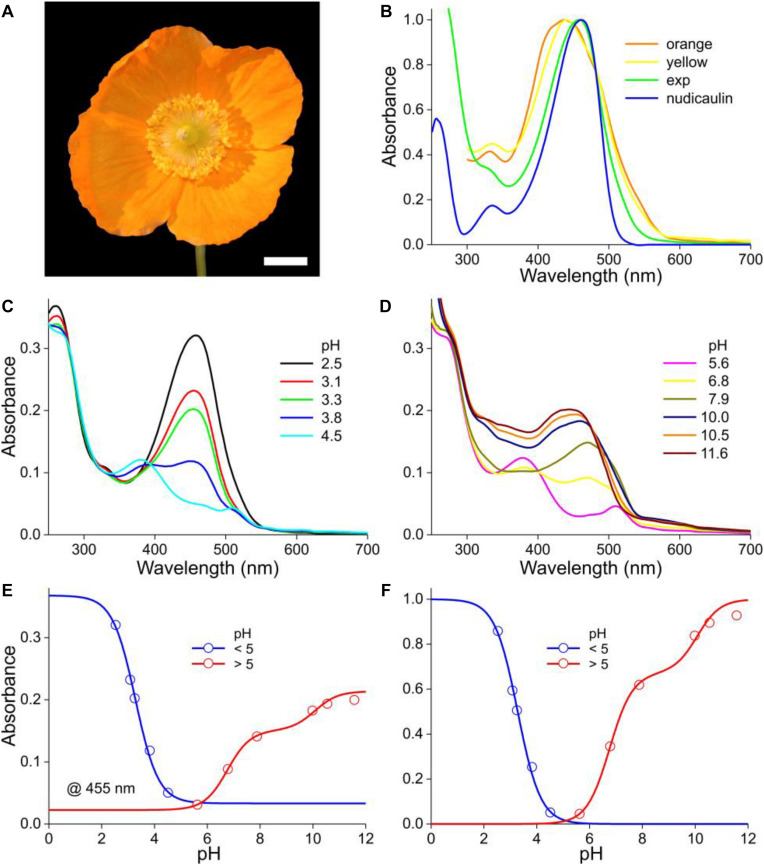
The Welsh poppy *M. cambrica* and pH-dependent absorbance spectra. **(A)** A flower; scale bar 1 cm. **(B)** Normalized absorbance spectra of petals of orange and yellow morphs (from van der Kooi and Stavenga, 2019), the normalized spectrum of panel **(C)** for pH = 2.5 (exp), and that of nudicaulin (from Dudek et al., 2016). **(C)** Absorbance spectra for pH < 5. **(D)** Absorbance spectra for pH > 5. **(E)** Absorbance values derived from panels **(C,D)** at 455 nm (symbols), fitted with logistic curves. **(F)** The data of **(E)** with background subtracted and normalized.

The Welsh poppy, *M. cambrica*, features bright yellow or orange flowers. The normalized absorbance spectra of intact yellow and orange flowers have very similar shapes (Figures 5A,B), suggesting the presence of the same pigment, but with the yellow flowers having a lower concentration (Figure 6B of van der Kooi and Stavenga, 2019). Yellow *M. cambrica* flowers are colored by nudicaulins (Tatsis et al., 2013), and therefore Figure 5B shows the absorption spectrum of nudicaulin glycoside isolated from *Papaver nudicaule* (from Dudek et al., 2016). It indeed closely resembles the absorbance spectrum of methanol extracts of orange flowers at low pH (Figure 5B, exp).

As in the previous cases, the absorbance of *M. cambrica* flower extracts decreases steadily with increasing pH (Figure 5C). Similar as in *P. nudicaule* petal extracts, between pH = 4 and 7 a minor absorbance band at 510 nm arises (Dudek et al., 2018). However, with increasing alkaline pH the absorbance band shifts again to lower wavelengths and rises in about the same wavelength range as that of the acid pigment state. The resulting absorbance spectra peak in the blue wavelength range at 460 nm at strongly acidic pH, at 510 nm at weakly acidic and at 445 nm at alkaline pH. To investigate the pH-dependence of the three phases, we assessed the absorbance values of the spectra of both Figures 5C,D at 455 nm. Fitting the pH < 5 data with a one-component logistic function yielded pK = 3.3, and fitting the pH > 5 data with a two-component logistic function yielded pK = 6.6 and 10.0. The absorbance spectra (or their difference spectra) could not be approximated with a restricted set of absorbance spectra similar to the cases treated above. A separate, more extensive approach will be necessary to unravel the multiple participating components. We also analyzed the yellow form of *M. cambrica*. It suffices to note here that the absorbance spectra of extracts of yellow flowers showed a similar pH-dependence as that of the orange form, with only very slight spectral differences.

## Discussion

We have studied how floral pigment absorbance depends on pH in a few flowers with flavonoid and flavonoid-derived pigments. We chose two different poppy species that have a largely similar anatomy and backscattering but differ in pigmentation and coloration (van der Kooi and Stavenga, 2019). We furthermore investigated two *Mandevilla* varieties. Interpreting studies on cultivated plants from an ecological and evolutionary point of view needs caution, because it is hard to know what phenotypic traits have arisen in nature and have been selected by plant breeders. Nevertheless, cultivated plants can be useful to study specific optical properties, particularly when cultivated lines greatly vary in one specific trait. In the case of the studied *Mandevilla* plants, cultivation resulted in flowers of similar thickness and backscattering (two important aspects of flower coloration; van der Kooi et al., 2016), but with highly different types of pigmentation (Figure 4C). The two poppy species and the two *Mandevilla* varieties thus provide a valuable resource for studies on the fundamental pigmentation properties of flowers.

Absorbance spectra of methanol extracts generally corresponded well with absorbance spectra measured from intact petals, although slight differences in spectral shape were encountered in all studied cases. Detailed chemical analyses revealed that flowers contain a multitude of flavonoids. For instance, the flowers of the common corn poppy, *P. rhoeas*, contain several glycosides of the flavonols kaempferol and quercetin as well as of the anthocyanidins cyanidin and pelargonidin (Dudek et al., 2020). The diversity in the absorbance spectra thus is not surprising, as extracts differ in solvent and concentration from the vacuolar conditions and may thus alter associative effects between pigments and solvent. Glycosylation as well as acylation can distinctly modify the absorption spectrum of the flavonoids (Giusti et al., 1999; Dudek et al., 2016). Furthermore, substantial spectral shifts can be exerted by co-pigmentation (Mazza and Brouillard, 1990).

Considerable insight has been assembled concerning the biosynthesis, the genetics, and the evolution of flower pigments, especially of the carotenoids and anthocyanins (Koes et al., 1994; Mol et al., 1998; Grotewold, 2006; Rausher, 2006; Glover and Martin, 2012; Campanella et al., 2014), but detailed studies on the pH dependence of pigment absorbance spectra, especially of complex pigment compositions, are scarce. The absorbance spectra of the cyanidin and pelargonidin derivatives encountered in the extracts of *P. dubium* flowers severely depend on pH. For pH < 5, the pH dependence is well described by a declining, single-component logistic function with pK ∼ 3. This conforms to the general characteristic of flavylium compounds, where the distinctly absorbing cation converts into colorless hemiketal and *cis*- and *trans*-chalcone forms (Mazza and Brouillard, 1990; Pina et al., 2012). At higher pH various anionic quinonoidal bases are created (Mazza and Brouillard, 1990; Pina et al., 2012; Rakić et al., 2015; Dangles and Fenger, 2018; Sigurdson et al., 2018), which process can be approximated with a two-component logistic function. The differences in the absorbance spectra of Figures 2, 3 in the long-wavelength range suggest that the absorption spectra of the quinonoidal bases depend on being cyanidin- or pelargonidin-based derivatives.

The absorbance spectra of the *M. sanderi* “Red Sundaville” flower extracts show in the visible wavelength range a similar pH-dependence as that of the cyanidin-dominated *P. dubium* (Figures 3A,B, 4E,F), but the spectra in the UV-wavelength range differ considerably. We, therefore, investigated the flowers of the *M. sanderi* “White Sundaville,” because they contain only UV-absorbing pigment (Figures 4A,D), having at low pH an absorbance peak wavelength of 325 nm and at high pH 382 nm (Figure 4J). We tentatively attribute this to flavonol, because its pH-dependence follows a two-component logistic function that is very similar to that of the anthocyanin. The same (or very similar) flavonoid pigment appears to exist in the Red Sundaville (Figure 4O).

Flavonoids have been identified as colorants in numerous flowers (Iwashina, 2003; Zhao and Tao, 2015). For instance, in methanol extracts of *Dipladenia martiana* flowers, the flavonols quercetin and kaempferol and several related components were identified (de Carvalho et al., 2001). The spectral analysis of the investigated *Mandevilla* (*Dipladenia*) flowers suggests that the different types of pigment (flavonols in the White morph versus flavonols and anthocyanins in the Red morph) behave similarly with regard to pH changes.

The Welsh poppy, *M. cambrica*, is a special case as its flowers are colored by nudicaulins, an unusual group of indole alkaloids, which are generated by combining anthocyanins with an indole (Tatsis et al., 2013; Dudek et al., 2016, 2018; Devlin and Sperry, 2020). We note that the pH-dependent processes of *M. cambrica*‘s nudicaulin can be described by logistic functions with very similar pK-values as those of the anthocyanins (Table 1). The nudicaulin pigments were analyzed in great detail in the Iceland poppy, *P. nudicaule* (Tatsis et al., 2013; Dudek et al., 2016, 2018). Interestingly, the yellow stamens of the yellow flowers contained carotenoids and not nudicaulins (Dudek et al., 2016). Yellow flowers generally contain carotenoids, which is also the case with the *M. sanderi* flowers, where carotenoids are expressed in the flower tube (see Figures 4A,B). In the yellow/orange-colored flowers of *M. cambrica* we did not obtain convincing evidence for anthocyanins.

**TABLE 1:** Summary of the pK-values obtained for the pH-dependent processes in the studied flower extracts.

**Flower**	**pH < 5**	**pH > 5 (λ < 500 nm)**	**pH > 5 (λ > 500 nm)**
*P. dubium* 1 (Figure 2)	3.1	6.6/9.0	6.0/9.1
*P. dubium* 2 (Figure 3)	3.2	7.3/9.4	7.1/9.0
White *M. sanderi* (Figure 4)	–	6.9/8.8	–
Red *M. sanderi* (Figure 4)	3.1	6.4/8.4	5.7/8.8
*M. cambrica* (Figure 5)	3.3	6.6/10.0	–

*For pH < 5, a one-component logistic function was fitted to the absorbance changes, while for pH > 5 in both the short- and long-wavelength ranges a two-component logistic function was used.*

The absorption spectra of floral pigments strongly depend on pH, which has severe consequences for flower coloration. The important question then is to know the vacuolar conditions in the flowers. Measurements of the pH of flower epidermal cells yielded values varying between 2.5 in a begonia cultivar and 7.5 in morning glory cv. Heavenly Blue (Stewart et al., 1975). Curiously, in the epidermal cells of flowers whose only anthocyanins were cyanidin glycosides, the associated colors widely varied, between strong pink, deep red, purple, and even moderate blue, whereas the pH ranged from 3.1 to 5.5 (Stewart et al., 1975). In this pH range, the anthocyanins’ absorption spectra have similar shapes. The absorption amplitude is at most half of the maximal value, and at higher pH-values it further diminishes. A solution to the loss in absorption upon increasing pH is co-pigmentation, which stabilizes the acidic pigment state and can modify the spectral absorption (Mazza and Brouillard, 1990).

The anthocyanins are concentrated in the epidermal cells’ vacuoles, and therefore a crucial factor determining the flower’s color is the vacuole’s pH, which is highly dependent on an H^+^-ATPase (Verweij et al., 2008; Yoshida et al., 2009). Minor changes in pH can cause major changes in coloration. Indeed, a pH change from 3.3 to 4.0 in the vacuoles in *Hydrangea macrophylla* sepals makes the color shift from blue to red (Yoshida et al., 2003). A similar color change occurs in the morning glory cv. Heavenly Blue during the flower-opening period, but it is here due to an unusual increase in vacuolar pH from 6.6 to 7.7 acting on a tricaffeoylated anthocyanin, the heavenly blue anthocyanin (Yoshida et al., 2009). We conclude that for the flowers studied in the present paper, and probably for anthocyanin- and flavonol-based colors more broadly, vacuolar pH crucially determines the pigment absorbance spectrum.

An intriguing question is how vacuolar pH, pigment absorbance spectra, and structural aspects of flowers are tuned to optimize visibility to (local) pollinators. Previous studies demonstrated that regional differences exist in the coloration of *P. rhoeas*, which can be linked to the color vision of local pollinators, beetles in the Middle East, and bees in Europe (Dudek et al., 2020; Martínez-Harms et al., 2020). Also, *Mandevilla* flowers are pollinated by different groups of pollinators, including bees, butterflies, and moths (De Araújo et al., 2014; Rubini Pisano et al., 2019), which have different visual systems (van der Kooi et al., 2021). When considering the ultimate question of how flower colors are tuned to the visual system of their pollinators, disentangling the different structural as well as pigmentary aspects that create the flower’s coloration is important.

We previously showed that in addition to pigments, the reflection and scattering properties of the flower structures also modify the coloration, and that scattering and pigmentation properties may be tuned for visual signaling to pollinators (van der Kooi et al., 2016, 2019; Stavenga et al., 2020). The emerging picture is that flowers have a wide gamut of possibilities to tune their coloration. A simple possibility is demonstrated by the yellow and orange flowers of *M. cambrica* that merely differ in concentration of essentially the same pigment (Figure 6B of van der Kooi and Stavenga, 2019). That subtle pH changes can greatly change the absorption spectrum of common flower pigments is important for studies on flower color evolution. The flower’s absorbance can be further modified by other factors as co-pigmentation, self-association, and metal complexation. As flowers are the advertisement flags for pollinators, the spectral properties of the floral pigments are presumably tuned to optimize visibility. In comparison, the photoreceptor spectral sensitivities of the color vision systems of bees and birds are highly constrained to virtually fixed spectral values (Hart and Vorobyev, 2005; van der Kooi et al., 2021). Plants thus have evolved a much greater flexibility in tuning the display of their flowers than the flexibility their pollinators have in adjusting their color discrimination system.

## Data Availability Statement

The original contributions presented in the study are included in the article/supplementary material, further inquiries can be directed to the corresponding author/s.

## Author Contributions

DS performed the analysis, made figures, and wrote the manuscript. HL executed the chemical analysis. BD and CK gave crucial input to the final manuscript. All authors contributed to the article and approved the submitted version.

## Conflict of Interest

The authors declare that the research was conducted in the absence of any commercial or financial relationships that could be construed as a potential conflict of interest.
